# Diagnosis of Canine Cognitive Dysfunction Syndrome: A Narrative Review

**DOI:** 10.3390/vetsci12080781

**Published:** 2025-08-20

**Authors:** Claudia Vitturini, Matteo Cerquetella, Andrea Spaterna, Marilena Bazzano, Andrea Marchegiani

**Affiliations:** School of Biosciences and Veterinary Medicine, University of Camerino, Via Circonvallazione 93/95, 62024 Matelica, Italy

**Keywords:** cognitive dysfunction, clinical diagnosis, biomarkers, canine dementia

## Abstract

Many recent progresses in the overall quality of life have allowed for an increase in life expectancy, both in humans and in dogs. In addition, long-lived individuals may develop neurodegenerative disorders, and one of the most important in human medicine is Alzheimer’s disease (AD). In veterinary medicine, the AD counterpart is Canine Cognitive Dysfunction Syndrome (CCDS), which, generally, affects elderly dogs from 8 years of age. These cognitive disorders are becoming frequently encountered conditions and, despite researchers’ attention towards pathogenesis, treatment and diagnosis, more efforts are required to outline which clinical and laboratory evaluations must be carried out to reach a presumptive antemortem diagnosis of CCDS. The biggest need is the establishment of standardized protocols and guidelines for a correct clinical and diagnostic approach towards dogs with clinical signs referrable to CCDS. In this narrative review, we examined the up-to-date scientific literature on the topic, focusing our attention on sensitive and reliable markers for clinical antemortem CCDS diagnosis. Even if some parameters analyzed are interesting and promising, more investigations are needed to confirm the results obtained so far. This is crucial because a correct diagnosis is fundamental to determine the best treatment and, thus, to guarantee animals’ health and welfare.

## Simple Summary

Canine Cognitive Dysfunction Syndrome is a neurodegenerative disorder affecting elderly dogs (~8 years old) and the relationship between animals and their owners, who generally find difficulties in the management of their pets. In recent decades, the incidence of this disorder has been increasing but antemortem diagnosis is still a challenge for veterinarians because of the lack of a well-defined diagnostic protocol. The aim of this article is to give a general overview of the clinical procedures, tests, markers and protocols which researchers have proposed so far as valid elements for diagnosing this subtle disease, even if more studies are needed. The early individualization and the application of the correct treatment are crucial factors for animals’ and humans’ wellness.

## 1. Introduction

The technological and economic growth, the pharmaceutical and nutritional advances, and the progresses in human and veterinary medicine are the main factors which have contributed to the longer lifespan of humans and companion animals. Moreover, people are more aware about the welfare and well-being of their pets [1,2]. Nowadays, the senior population of both human and animals is exponentially increasing, as is, parallelly, the incidence of neurodegenerative diseases without available effective therapeutical solution counterparts [3,4], often representing the cause of serious frustrations for owners and veterinarians [5]. One of the most important neurodegenerative disorders in humans is Alzheimer’s disease (AD), which has a huge impact on the quality of life of affected people and their relatives [6,7]. In veterinary medicine, dogs, and less frequently cat and horses, can show a syndrome superimposable to human AD called Canine Cognitive Dysfunction Syndrome (CCDS). It is considered the canine counterpart of human Alzheimer’s disease [8] since many neuropathological changes are shared between CCDS and AD [9]. Also known as “canine dementia”, CCDS is a chronic and progressive neurodegenerative disorder affecting elderly dogs, generally starting from 8 years of age [4,10,11]. Interestingly, immunosenescence and inflammaging are also described in dogs [12]. The age of onset of clinical signs is different depending on the breed. Indeed, the cutoff for senior dogs is lower in large dogs (6–7 years) compared to small dogs (8–10 years) [3,4]. It has been ascertained that CCDS prevalence increases with increasing age [8]. About 19% of dogs aged 11 to 13 years are affected by CCDS and the prevalence can increase up to 45.3% in 15-year-old dogs [13]. Moreover, worsening symptoms are present in up to 68% of animals over 15 years old [14,15]. The prevalence is underestimated, with less than 2% of dogs receiving a diagnosis for CCDS [16]. The symptoms are often mistaken with typical tracts of the aging process and there is still a lack of well-established diagnostic tools, mainly due to the fact that elderly dogs, like humans, do not show their rate in cognitive impairment in a standard way, and great variability and individuality also make diagnosis more challenging [17]. Some dogs can face the aging process without it affecting either their routine or their relationship with owners. Conversely, other dogs could develop dementia, which may lead to several consequences [18], especially regarding the relationship with their owners, which often perceive this situation as a significant burden that might deteriorate the pet–owner relationship [19]. As stated above, many factors can interfere with the clinical manifestation of CCDS and, in general, of neurodegenerative disorders. The lack of a current standard diagnostic protocol is probably due to this great variability and to other factors which will be analyzed in this review. It is fundamental to diagnose CCDS as soon as possible, because only with an effective therapy is it possible to try to slow down the neurodegenerative process and to improve pets’ and owners’ quality of life. The aim of this narrative review is to understand the different methods and tools proposed until now for the clinical antemortem diagnosis of Canine Cognitive Dysfunction Syndrome.

## 2. Materials and Methods

For this literature review, the PubMed and Scopus repositories were checked to retrieve scientific articles using the following keywords: “canine cognitive dysfunction syndrome”, “diagnosis canine cognitive dysfunction syndrome”, “canine cognitive dysfunction”, and “diagnosis canine cognitive dysfunction”; no restriction of time was applied.

## 3. Pathogenesis: Similarities and Differences with Human Alzheimer’s Disease

One of the major risk factors related to the development of neurodegenerative disorders is the aging process itself [9]. Sex has been described as a potential risk factor, as that female dogs seem to be more prone to develop CCDS within the elderly dogs’ population, similarly to what happens in human AD [20]. Several theories tried to explain the causes of aging, but the most accepted one seems to be the “free radicals’ theory of aging”: during aging, all tissues and organs progressively lose their function because of the accumulation of free radicals’ damage to macromolecules (i.e., proteins, lipid, DNA and carbohydrates) [17,21]. When antioxidant systems are not able to limit free radicals’ actions, these can bind the major cellular molecules, leading to their irreversible structural and functional modifications [21]. As the average oxygen consumption is higher in the brain (20% of the total body), this is one of the tissues most susceptible to free radicals’ action, as well as due to high amounts of polyunsaturated fatty acids and a lower level of antioxidant systems [3]. In CCDS, as during AD, there is evidence of increased free radicals’ and DNA damages, decreased antioxidant systems and mitochondrial activity, as well as an increase in inflammation and altered neurotransmitter production (e.g., norepinephrine, acetylcholine, dopamine and GABA) [21]. Other changes include the major production of acetylcholinesterase in association with a cholinergic fall, the increased activity of monoamine oxidase B with increasing catalysis of dopamine and production of free radicals, the decline in cholinergic tone, the decreased density of muscarinic receptors in brain and an increased concentration of potassium, lactate and pyruvate in the cerebrospinal fluid [6,10]. Within the brain, other main patterns of neurodegeneration are represented by amyloid beta (Aβ) deposition in extracellular parenchyma and hyperphosphorylation of tau protein, a protein normally associated with microtubule [22]. Aged dogs spontaneously deposit human-type Aβ peptide, and it is one of the most important reasons why dogs are considered the best mammalian spontaneous model of aging for the study of AD [23]. β-amyloid protein is a product of the amyloid precursor protein’s (APP) degradation [3], which undergoes a double cleavage by two endoproteases: β-secretase and γ-secretase. The final result of these two enzymes’ action is the Aβ in two main forms: Aβ-42 and Aβ-40 [22]. The Aβ 40 amino acids long (Aβ-40) seems to be more abundant (80–90%) than the hydrophobic Aβ-42 form (5–10%) [2]. In both AD and CCDS, Amyloid-β plaques are mainly composed of Aβ-42 peptides, which strongly tend to form aggregates and to precipitate, leading to a decrease in Aβ levels both in cerebrospinal fluid (CSF) and plasma, compared with elderly healthy dogs [11]. The deposition of Aβ protein involves different canine brain areas, such as prefrontal and parietal cortices, hippocampus and the occipital cortex, until the formation of plaques: these aggregations seem to also be correlated with increasing apoptosis processes and, thus, neuronal loss [3,24]. Aβ deposition may also occur years before the onset of clinical signs, leading to a progressive cognitive impairment until the most severe stages of dementia [6,25]. In particular, the increasing extent of Aβ deposition in the cerebral cortex seems to be positively correlated with declines in cognitive functions both in laboratory dogs and in aged pet dogs [26], leading to learning and memory decreases [22,23]. However, there are also contrasting findings about Aβ deposition [13]. In some cases [4,22,27], it seems to be tied to cognitive dysfunction, but other authors [28] found no correlation between Aβ deposition and failures in cognitive task’s performances in laboratory beagles [29]. The confirmatory diagnosis of CCDS is still a postmortem diagnosis: Aβ plaques and other neuropathological features at immunohistochemical analysis confirm the presence of the neuropathological process. Another hallmark for human AD is the formation of neurofibrillary tangles (NFTs) from hyperphosphorylated tau protein [13]. Some authors argue that tau protein phosphorylation is not verified in dogs [30], while others described this event in dogs’ brain, especially in the hippocampal region [13]. Interestingly, Dewey and Rishniw have drawn attention to the theory that in the development of human AD, periodontal diseases play an important role [31]. In particular, the alteration in the oral microbiome with the consequent increase in Gram-bacteria leads to a chronic status of inflammation. Inflammation itself may be a crucial factor for AD onset: pathogenic bacteria may cause the disruption of the blood–brain barrier and the response to the inflammatory process may cause the deposition of neurotoxic β-amyloid in brain. The positive association between periodontal diseases and AD has been demonstrated in humans, but in veterinary medicine, this evidence must be more investigated [31].

Combining knowledge of the two diseases in the two species could lead to mutual benefits in the One Health perspective [32].

## 4. Diagnosis of CCDS

In clinical practice, a specific and reliable protocol for the antemortem diagnosis of CCDS is still lacking [33]. Some authors [4,11,34,35,36] proposed the following clinical steps to be carried out during the first evaluation of patients with behavioral alterations: medical history collection, physical, neurological and orthopedic examinations, complete blood tests (including blood count, chemistry and thyroid panel), evaluation of blood pressure, thoracic radiographs, and abdominal ultrasound. If the results of these exams are normal, the protocol should proceed with MRI examination to exclude structural changes and a cerebrospinal fluid analysis to rule out inflammations, neoplasia and infections [11], and then the completion of screening questionnaires by the dogs’ owners [35]. In human and veterinary medicine, many efforts have been carried out to find out markers able to predict AD and CCDS disruption, but these markers are still lacking [9,34]. Early diagnosis of neurodegenerative disorders is needed because therapeutic interventions would be more effective at the initial stages [30]. In recent decades, the focus on CCDS has been growing and several researchers have decided to face this diagnostic challenge through investigations for methods like tests, biomarkers and other possible parameters for CCDS diagnosis (Table 1). Regarding behavioral changes, the most common are summarized by the acronymous DISHA(A): **D**isorientation in familiar environments, alterations in social **I**nteractions (both with other animals living in the same house and with owners), **S**leep–wake cycle alterations, increasing **H**ouse-soiling, and alterations in **A**ctivity levels [3]. For the latter “A”, someone also indicates increasing **A**nxiety and, in other cases, increasing **A**ggressivity, which has sometimes been the most reported sign [3]. Unfortunately, these clinical signs are very unspecific, and it is fundamental to understand if they are primary due to CCDS or consequences of other systemic conditions. However, in elderly patients, there could be a combination of these two possibilities [6]. There are a lot of pathological conditions (e.g., pain, metabolic disorders, organ’s failure, brain neoplasm, endocrinopathies, gastrointestinal and dermatologic diseases, sensory declines) common in aging pets that can deeply affect behavior [11,37]. Moreover, disorientation could be the consequence of hearing loss, visual loss, or any other senses’ disorders [3].

### 4.1. Questionnaires

Owner-based questionnaires are very useful tools to primarily start the difficult path to reach a CCDS diagnosis, but the results obtained must be supported by the presence of clinical signs and clinical evaluation to exclude the coexistence of other disorders [8]. Also, the bias related to the “predisposition” and subjectivity in interpreting the signs of the person who fills it in is a factor present in every questionnaire (Table 2). These are screening tools commonly used to assess dogs’ behavioral and cognitive alterations over time [18]. Behavioral signs are often the only features characterizing a cognitive decline in animals and humans, and the early identification of these alterations represents the main opportunity to try to prevent and slow down neurodegeneration [24,32]. However, the identification and assessment of the early onset of behavioral alterations is extremely challenging [15], as they often go unnoticed [34], and should also be interpreted considering the observer’s subjectivity. The major consequences of cognitive decline, in terms of severity, affect learning ability, memory, and adaptability to new situations, and other skills such as problem solving, attention, visuospatial function, socialization, decision-making ability, and the capacity to obtain and process information [6,24,29]. However, while impairments in learning and memory are considered the main hallmarks of cognitive decline, many subjects can show minimal or even no alterations in these items [18,50]. Owners have a pivotal role in the recognition of pets’ behavioral abnormalities, and their education and awareness should be a priority for veterinary clinicians through educational tools, handouts, and web links on senior care and CCDS [6]. A standard delineation of behavioral signs in CCDS patients to help distinguish from normal aging is still lacking [51] and veterinarians must rely on the owners’ evaluations of dogs’ behavior, but more objective, unbiased and quantity-reliable parameters are needed.

The most widely used questionnaires are the Canine Cognitive Dysfunction Rating scale (CCDR) and the CAnine DEmentia Scale (CADES). Both questionnaires have been validated using appropriate psychometric evaluations and these tools have some differences, e.g., the different stages of CCDS captured [35]. The CADES questionnaire [53] seems to give a better idea of the different stages of CCDS, to identify cognitive impairment earlier and to monitor the progression of this disease over time if compared with CCDR. Moreover, the combination of the CADES questionnaire and the evaluation of plasma biomarkers may be better at predicting CCDS in its early stages [11]. The CADES questionnaire concerns 17 items divided into 4 domains and permits the classification of dogs into “mild”, “moderate” and “severe” impaired subjects based on behavioral alteration stages [36]. CCDR was designed to find a clinically and ethologically valuable screening tool for CCDS diagnosis [51]. In this questionnaire, there are 13 items concerning behavioral problems related to apathy, memory, locomotion and impaired olfaction. The score for each item can range from 1 (never) to 5 (>once a day), and the total final score is used to distinguish non-impaired dogs from impaired ones. The CCDR scale is a valid tool to assess the level of cognitive impairment and a total final score > 50 accompanied by a veterinary assessment seems to be diagnostic for CCDS in veterinary practice. Even if the diagnostic accuracy of CCDR scale is high (~98.9%), it seems to be more reliable when behavioral alterations are severe [48,51]. Another questionnaire was designed by Kiatipattanasakul et al., including items about posture alterations [52]. In fact, it is very common to see gait and posture alterations and alterations in perception in humans affected by mild cognitive impairment (such as motoric disorders, altered posture, and rigidity), which is considered a middle stage between normal aging and slight-to-mild dementia. To better understand the possible relationship between physical disturbances and CCDS, Ozawa et al. created another questionnaire divided into three sections about general information, physical disturbances and the CCDR [48]. The results showed a correlation between physical disturbances (smell disturbances, vision impairments, tremors, swaying or fallings) and the CCDR, especially for vision impairment (present in more than 90% of CCDS dogs), according to human medicine, where ocular tests could be used as potential biomarkers for an early diagnosis of AD. In addition, in human medicine, smell disturbances are related to neurodegenerative disorders, and it seems that dogs can also develop similar olfactory perception alterations [54]. A study suggests physical alterations as predictive clinical tools for CCDS diagnosis [48,52]. Olfactory dysfunction in CCDS dogs was also previously observed by Schütt and collaborators [8]. Moreover, in another study [27], three different questionnaires called “Questionnaire A” [55], “Questionnaire B” [52] and “Questionnaire C” [56] were used together to evaluate the correlation between the questionnaires’ scores and brain alterations in elderly dogs (i.e., cortex atrophy, macrophage accumulation, myelin breakdown, amyloid deposition, and accumulation of oxidative damage). The results suggested that these three questionnaires taken together showed a higher correlation with the pathological features investigated, showing that using only a single questionnaire with few items may lead to an incorrect diagnosis of cognitive decline [27]. More recently, the CADES questionnaire was used with few changes to identify dogs with presumptive CCDS [11]. The authors of the study found that in mildly and moderately impaired dogs, the category with the highest score was the sleep–wake cycle, and in severely impaired dogs, the highest score was related to spatial orientation [11]. Fefer et al. tested the use of both questionnaires (CADES and CCDR) to classify dogs’ impairment, and the results showed that the CADES questionnaire permits a better classification of the several stages of CCDS (severe, moderate, mild and normal), while CCDR helps to distinguish between individuals at high risk of developing CCDS and normal individuals. Interestingly, one dog classified as severely impaired with the CADES questionnaire was classified as normal aged with the CCDR one, and two dogs considered moderately impaired and one dog considered mildly impaired with CADES were classified as “at risk” by CCDR [35]. In the effort to also evaluate aging cognitive alterations in different places than laboratories, Le Brech et al. [14] designed a cognitive test for the monitoring of dogs living in a home environment: the Canine Cognitive Assessment Scale (CCAS) in association with the Practical Cognitive Test (PCT). The latter can also be used with dogs not living in a home environment. CCAS is a questionnaire that has been modified from the others already known in the literature; it is divided into 6 groups (Disorientation, Sleep–wake cycle, Anxiety, Activity level, Learning and memory and Social interactions), which include 17 items to be addressed by owners on a 0 (never) to 3 (almost every day) scoring system. After filling out the questionnaires, the dogs could be assessed with the PCT, which consists of two tasks: discrimination learning and reversal learning. The authors hypothesized that the results of the PCT would predict the CCAS final score, but there were no significant correlations [14].

Haake et al. investigated the relationships between the Canine Dementia Scale (CADES), the Canine Cognitive Dysfunction Rating Scale (CCDR), and the Canine Cognitive Assessment Scale (CCAS) [37]. Another questionnaire was tested, the Canine Behavioral Assessment and Research Questionnaire (C-BARQ), which is not designed for the detection of dementia but for behavioral problems in general [37]. The authors found a strong correlation between CADES and CCAS, highlighting their efficacy in the prediction of the progression of dementia from a mild case to a more severe one (especially for the items “social interaction” and “activity” in CADES and CCAS, respectively). The CCDR is more focused on the frequency of behavioral signs; it showed a weaker correlation with the other two questionnaires, but it was found to be very useful for dogs with severe cognitive impairment. The C-BARQ questionnaire was used to investigate items not covered by the other questionnaires which can affect dogs with CCD: fear, trainability, pain sensitivity and anxiety. The C-BARQ domains assessing fear were correlated with higher CCD scores related to mild states of dementia. This paper confirms that many factors can influence the choice of the questionnaire to use and that more studies are needed to understand which questionnaire is more suitable for early detection of CCD [37].

### 4.2. Cognitive Tests

Even if owner-based questionnaires are very useful tools for the identification of behavioral changes, more objective and diagnostic options have been developed [47].

Several cognitive and psychological tests were taken from human medicine and then modified to be applied to dogs in a laboratory setting. Some examples are the Delayed Non-Match Position, and oddity, object, size, landmark and picture discrimination tasks [6]. However, these tests protocols were standardized on Beagles and on laboratory setting, resulting in very long and expensive procedures [18]. Other neuropsychological tests have been developed and validated to assess the cognitive function in dogs, such as discrimination learning, reversal learning, spatial memory and oddity learning, and Delayed Non-Match Position, which are standard cognition tests used for dogs and should be conducted in a laboratory setting [25,50]. The Delayed Non-Match Position test evaluates the short-term visuospatial memory. In the first step, the dog (if we consider dogs) should displace only the object covering the food reward (marked with a letter), choosing among two other possible objects. After a short delay, the second phase starts with the same objects of the first phase, with the marked object covering the food reward present in the first identical position. The dog can choose the right object and, so, win the food reward or can make errors choosing one of the other objects [6]. Dogs’ failures in learning tests, discrimination learning, reversal learning, and spatial learning have strongly positive correlations with higher levels of Aβ depositions [57]. Studzinski et al. decided to evaluate the visuospatial function in cognition-impaired dogs because, in people affected by AD, memory and spatial learning are severely compromised and these impairments can be observed before any other cognitive alterations. The authors tested dogs using two versions of the Delayed Non-Match to Position (DNMP) task, finding out that visuospatial declines can be observed in dogs of 6 years of age. Considering that CCDS can be observed in dogs aged around 8 years old, these cognitive tools could be useful for the individualization of early signs of CCDS in dogs [46]. Borghys et al. adopted different cognition tests for their evaluation (reward and object approach, discrimination and reversal learning, and Delayed Non-Match Position), trying to correlate Aβ-42 levels in canine CSF with presumptive CCD [25]. The results allowed the authors to positively correlate errors in DNMP, learning and reversal learning with CSF Aβ-42 levels when all dogs were considered, highlighting that the DNMP task is affected early in canine aging [25]. Other cognitive tests are represented by food-searching tasks (more reliable) and problem-solving tasks, the advantages of which are quickness and the possibility to be performed for clinical use [10]. González-Martínez et al. used these two tests in a clinical setting [47] with the aim of evaluating the possible correlation between the tests results, the age of dogs, and their cognitive impairment stage. The cognitive status was firstly established with the use of a non-validated questionnaire. As a result, the food-searching task was useful to differentiate dogs with different levels of severity of CCD, but it could not differentiate normal-aging dogs from mildly impaired ones. The results of the problem-solving test were correlated with the dogs’ age: younger dogs obtained better results than the older dogs, but there was not a significant correlation between this test and the presence of CCDS [47]. Several years later, Romano et al. [49] studied the responses of elderly dogs, with and without cognitive impairment (control group), to the food-searching task. The dogs previously underwent clinical evaluations, blood and urine tests, and abdominal ultrasounds. Moreover, the owners were asked to fill out a questionnaire with 18 items concerning the signs included in the acronymous DISHA(A). From the results, the authors concluded that behavioral changes were positively related to advanced age, but the food-searching task could not discriminate dogs with cognitive impairments from those without cognitive deficits. However, impairment negatively affected dogs’ performances [49]. Chapagain et al. focused on dogs’ cognitive and behavioral aspects, which are determinant in the relationship with their owners (i.e., independent and social problem solving, trainability and learning), called by authors “individual control processes” and “social control processes”, using a Modified version of the Vienna Canine Cognitive Battery (MVCCB) together with the Vienna Dog Personality Test [58]. In particular, the fields included in this evaluation were exploration, picture viewing, food choice, separation, greeting and playing, memory test with distraction, detour, attention, novel action, manipulative persistency and, finally, clicker training for eye contact. From their results, the factor most influenced by cognitive impairment was the problem-solving one [18]. Recently, Hargrave et al. set up a new neuropsychological test to assess dogs’ cognitive impairment, focusing on social behavior, spatial memory and executive function [32]. This battery includes five spontaneous problem-solving tests to execute in two different sessions of 30–60 min. In the first session, dogs perform two memory tasks (delayed search task and a two-location task) and a sensory screen, while in the second session, a measure of human social interaction (with an unfamiliar experimenter) and two measures of executive functions (spatial reversal task and cylinder task) are included. The authors tried to assess a possible correlation between dogs’ cognitive abilities and body mass. The results suggest a general negative correlation between performances in cognitive tasks and aging, except for social interaction. The worse performances associated with aging are especially related to the delayed search task and spatial reversal task. No significant evidence was found for body mass and cognitive impairment [32].

### 4.3. Biomarkers in Peripheral Blood

To detect and to slow down β-amyloid deposition in the brain before it becomes irreversible, it is essential to determine reliable biomarkers [26]. Nowadays, diagnostic tools rely on screening biomarkers in blood and cerebrospinal fluid, but dog owners often do not agree with CSF sampling, and blood tests are the main screening possibilities [42].

Considering that Aβ-40 and Aβ-42 have been suggested in human AD as non-invasive peripheral biomarkers to distinguish cognition-impaired people from healthy ones with high sensitivity and specificity, these have also been investigated in dogs. Sampling was preceded by a clinical evaluation and it was carried out using a questionnaire divided into four categories (sleep–wake cycle, house-training and commands, disorientation and socio-environmental alterations) to obtain a score and to distinguish healthy and mild-to-severe cognition-impaired dogs. The results in dogs showed that Aβ-40 and Aβ-42 levels change during aging, with higher levels in the blood of young dogs compared to aged dogs, suggesting this decrease could be due to Aβ deposition in brain [35,42]. Moreover, the level of Aβ-42 was higher in mildly cognition-impaired dogs than unimpaired and severely impaired ones. These findings suggest a similarity between dogs and humans, with the possible investigation of Aβ-42 as a blood marker for cognitive impairment [26]. Schütt et al. tried to demonstrate a correlation between systemic levels of Aβ-peptides (Aβ-42 and Aβ-40) and CCDS using a commercially available ELISA sandwich kit [8]. The owners were asked to fill out the CCDR questionnaire to classify dogs into three groups: non-CCDS, mildly cognition-impaired, and CCDS. The analyses results highlighted a significant difference among the three groups, with higher plasma levels of Aβ-42 in the CCDS group and an increased plasma level of Aβ-42 and Aβ-40 being positively correlated with the CCDR score, apparently in contrast with what was previously stated above. They also did not find significant differences between other biochemical or hematological parameters, either in the concentration of cytokines, fibrinogen or C-reactive protein which were also evaluated [8].

It has been shown that plasma neurofilament light chains (NFLs) are considered biomarkers for the early detection and progression of neurodegenerative diseases in humans with AD [41]. The NFL levels in blood are very low and, in most cases, undetectable using traditional assays. It was also demonstrated that, as in humans, NFLs can be detectable in dog’s plasma samples, and that NFLs’ amount strongly increased in relation to increasing age, with higher levels present in the plasma of impaired dogs. A positive correlation between plasma NFL amounts and CADES scores in senior and geriatric dogs was indeed found [41]. Similarly, Fefer et al. evaluated the correlation between the plasma level of NFLs and the results of cognition tests and of questionnaires scores, demonstrating that increased plasma levels of NFLs were significantly associated with higher CADES total scores [35]. The NFL levels in CCDS dogs were also measured using an immunomagnetic reduction (IMR) technique, where NFL antibodies are fixed on magnetic nanoparticles, demonstrating higher plasma levels of NFLs in CCDS dogs compared to those without CCDS [42].

Another study [4] collected canine blood samples, and the biochemistry results showed that, even if still in the physiological range, there was a significant elevation in alanine aminotransferase (ALT), total protein (PT) and gamma glutamyl transferase (GGT) in mildly cognition-impaired dogs compared to young dogs and non-impaired aged dogs. Moreover, there was a decrease in the levels of chloride and sodium in impaired dogs compared to young and normal-aging ones (the dogs were divided into groups based on their scores obtained from the CADES questionnaire developed by the authors). The researchers also investigated the blood levels of other parameters evaluated in humans affected by AD, such as NFL, tau protein and Aβ-42, using Simoa technology (Quanterix). The results demonstrated that in impaired dogs, the NFL amount was significantly increased compared to normal-aging and young dogs, but the tau protein and Aβ-42 amounts were very similar in all three groups [4]. Significant results were also shown by another blood test, as elevated liver and renal dysfunction biomarkers in a CCDS group compared to normal-aging dogs (SDMA, creatinine, total protein, urea nitrogen, albumin, AST, ALT and GGT) were found. Also, the C-reactive protein values were higher in CCDS dogs if compared to the non-impaired group, while chloride and sodium levels were lower (below physiological range) in the CCDS group than in normal-aging dogs, similarly to what was previously reported [42].

Interestingly, decreased levels of vitamin E were found in brain tissue of dogs with CCDS and in human plasma of AD patients. So, another study by Fast et al. [40] aimed to investigate vitamin E levels in the plasma of dogs affected by CCDS, proposing it as a possible biomarker for this neurological disorder. The authors classified dogs into the non-CCDS group, the borderline CCDS group or the CCDS group depending on the scores obtained from the owners’ questionnaire, the so-called “Questionnaire C” designed by Rofina et al. [27]. As a result, no significant correlations between cognitive decline and vitamin E serum levels were found [40].

Kim et al. analyzed three blood biomarkers as early predictors of CCDS: retinol-binding protein 4 (RBP4), C-X-C-motif chemokine ligand 10 (CXCL10) and NADPH oxidase 4 (NOX4) [34]. All the dogs included in the study (n=85) were divided into four groups (normal; mild cognitive impairment; severe cognitive impairment; and CCDS) based on the scores obtained from the CCDR questionnaire. The above-mentioned biomarkers were tested using ELISA kits and the results showed a significant reduction in the expression of RBP4, CXCL10 and NOX4 in the plasma of dogs affected by CCDS compared with normal dogs [34].

Another interesting study by Yoon et al. aimed to evaluate the blood concentrations of Aβ40, Aβ42, NfL and of the glial fibrillary acidic protein (GFAP) [43]. The latter is supposed to represent a possible biomarker of neurodegeneration due to its tendency to increase after astrocytic damage and inflammation during human AD. All these biomarkers were investigated through the ELISA technique from blood samples obtained from dogs with presumptive CCDS after the owners filled out three validated behavioral questionnaires (CADES, CCDR and CCAS). The results revealed a positive correlation between age and CDS severity, with older dogs affected by more severe cognitive impairment. Concerning the blood biomarkers, no significant differences were found in the Aβ40, Aβ42 and Aβ42/Aβ40 ratios between healthy dogs and dogs with CCDS (in contrast with previous studies). Serum NfL, instead, showed a strong positive correlation with CDS scores: higher levels of NfL were found in dogs with severe cognitive impairment. GFAP did not show significant variations between healthy dogs and cognition-impaired dogs despite the decreasing trend. This study confirms serum NfL as a possible reliable blood biomarker for the investigation of CCDS [43].

### 4.4. Biomarkers in Cerebrospinal Fluid

In humans, metabolic substrates as pyruvate and lactate increase in the cerebrospinal fluid (CSF) during AD [39]. These molecules, together with α-ketoglutarate, through HPLC with UV detection, and levels of glucose, total proteins and other ions involved in the energetic metabolism were also investigated in canine CSF in association with a newly developed test to characterize the progressive deterioration of memory, personality and cognition of dogs. The results showed that in severely impaired dogs, the levels of glucose, potassium, pyruvate and lactate were higher, suggesting an impaired glucose metabolism, which could be related to cognitive impairment [39].

As reported above, Amyloid-β plaques are mainly composed of Aβ-42 peptides, which strongly tend to form aggregates and to precipitate. Both in humans and dogs, the higher level of Aβ-42 depositions is followed by a lower level of Aβ-42 in the cerebrospinal fluid, and this happens years before the symptoms’ manifestation [25]. Borghys et al. investigated the CSF Aβ-42 levels in middle-aged dogs (from 1.5 to 7 years old) over a period of about 2 years, sampling CSF from the lateral ventricle. The intent was to evaluate the different amounts of Aβ-42 and if this difference was related to impairment verified with cognition testing. The Aβ-42 level in CSF was measured with the use of a multiplex immunoassay method and the results showed that a high level of CSF Aβ-42 early in life may be correlated with learning impairments, which likely precede cerebral amyloid deposition. So, this could be used as an early biomarker of cognitive impairment in dogs [25]. Aβ-42 levels in the canine cerebral area and in the cerebrospinal fluid of three dogs were also measured in association with owner-filled-out CCDR questionnaires for the evaluation of the correlations between analyses and the cognitive impairment score. Regarding CSF, researchers found a significant negative correlation between Aβ-42 levels and age, but also a significant positive correlation between Aβ-42 levels and CCDR scores [59]. Finally, in a recent case report, the presence of Aβ soluble peptides in CSF and in the blood of a 12-year-old Samoyed was evaluated through Sandwich ELISA (sELISA) and Western blotting. The results demonstrated different types of Aβ of soluble oligomers in CSF, with the prevalence of the two types Aβ-40 and Aβ-42, as happens in human AD [23]. However, the Aβ immunostaining described in this study was performed postmortem, showing diffuse plaques in the neocortex and hippocampal region of the cognitively impaired dog.

### 4.5. Magnetic Resonance Imaging (MRI)

The above-mentioned behavioral manifestations are associated with brain anatomical changes, which are not always easily observed using diagnostic imaging such as magnetic resonance [3,10,45], and that could also be observed in the brains of non-impaired, elderly dogs [10].

MRI is a non-invasive method that has multiple advantages as it permits researchers to determine objective parameters antemortem and it can allow veterinarians to rule out other pathological conditions. Typical MRI findings in CCDS dogs have already been mentioned above (brain atrophy, ventricular dilatation, enlargement of sulci, etc.), but their evaluation requires accurate measurements by expert clinicians.

The frontal cortex is one of the brain regions most vulnerable to the aging process and Aβ deposition in AD in terms of compromised integrity and the amount of Aβ deposits and decreasing volume [38,60,61]. The effects of aging on the frontal lobe, using in vivo MRI, were also evaluated in young and old dogs. Then, after the dogs’ death, the researchers evaluated the Aβ extension in the canine brain and found that there was a significant correlation between Aβ loads on the frontal cortex and its volume: higher Aβ loads were correlated with smaller frontal lobe volumes in aged dogs (in agreement with what happens in humans’ Alzheimer’s disease). Moreover, the same authors found that the frontal lobe volume increased until the dogs were 7 years old, and then remained stable until it started significantly decreasing during aging. Also, the hippocampal showed a significant reduction in its volume, suggesting this area is also vulnerable to the aging process (in this study, the decline was observed to start at ~10 years of age) [38].

The thickness of interthalamic adhesion (IA) was measured with MRI (in a transverse plane) to understand if it could be used as a parameter for brain atrophy in dogs. The results demonstrated a negative correlation between IA thickness and aging, and a positive correlation with body weight (even if weak). So, IA thickness was lower in cognition-impaired dogs compared to normal-aging ones, suggesting this parameter as a potential marker for brain atrophy [44]. Other authors considered the measurement of the IA as a possible marker for diagnosis of CCDS because a significant decrease in this cerebral structure in CCDS dogs was previously demonstrated. To avoid biased results between smaller dogs compared to bigger ones, the authors proposed the IA thickness/brain height ratio and lateral ventricle/brain height ratio as indicators for CCDS diagnosis. Measurements were conducted using computed tomography (CT) and MRI images and the results showed a significant difference of lower IA in the dementia group compared to young and normal-aging dogs. Moreover, the dementia group also had a significantly lower IA thickness/brain height ratio and lateral ventricle/brain height ratio compared to the normal-aging group, suggesting these measurements as possible values to quantify canine brain atrophy [45].

Finally, the hippocampal volume, using databases of MRI images of canine brains, was also investigated, comparing dogs diagnosed with CCDS and normal-aging dogs. It was demonstrated that CCDS dogs had a smaller hippocampal volume compared to normal-aging dogs, but the differences were very small and not sufficient to consider hippocampal volume as a valid parameter to discriminate CCDS dogs from normal ones. However, hippocampal volume reduction is not verified in normal aging and hippocampal atrophy is associated with cognitive impairment both in humans and in dogs [5,13].

Merbl et al. focused on the Blood–Brain Barrier (BBB) permeability through a particular technique called “subtraction enhancement analysis” (SEA) on MRI images: the authors hypothesized a changed BBB permeability in dogs with CCDS, and the aim of the study was to evaluate whether SEA could represent a diagnostic biomarker [16]. Shortly, SEA is a technique whereby an MRI sequence is digitally subtracted from the same identical sequence after the administration of a contrast medium. The results did not show significant differences in BBB permeability between healthy dogs and cognition-impaired dogs. However, the authors also investigated interthalamic adhesion diameters, which were found to be smaller in dogs affected by CCDS [16].

### 4.6. Electroencephalogram (EEG)

In human medicine, some electroencephalogram (EEG) findings have been successfully correlated with Alzheimer’s disease and considered early indicators for cognitive impairment [30]. Through power spectrum analysis and coherence analysis, it is possible to understand the meaning of each oscillation, which graphically represent the level of local extracellular potential and the synchronization among different brain areas. It has been demonstrated that alterations in oscillations are related to altered states of consciousness, cognition and memory. One preliminary study hypothesized the analysis of electroencephalograms as an additional tool for antemortem diagnosis of CCDS in dogs. Owners were asked to fill out the Rofina Questionnaire [27] and dogs were classified into normal, at risk of developing CCDS, and with CCDS using the Rofina dysfunction Score (RDS). Researchers demonstrated that dogs with behavioral alterations referable to CCDS showed brain electrical activity changes (i.e., loss of gamma coherence, increased joint Lempel–Ziv complexity [JLZC]). Furthermore, dogs at risk of developing CCDS showed higher alpha P3-P4 coherence than dogs with CCD, suggesting that alpha interhemispheric coherence should represent an early indicator of CCDS [30].

### 4.7. Monitoring Collars

Some authors recently investigated the potential of wearable collars able to track several types of information about physiological and behavioral changes compatible with CCDS, including vital signs such as heart rate, blood pressure, body temperature, and activity status of dogs, for their entire life. Those data could then be analyzed by a specific platform, and this tool could be useful for the individualization of changes referable to CCDS, but this needs to be further investigated [15,36].

## 5. Discussion and Conclusions

In this narrative review, we tried to summarize all efforts made in recent decades to diagnose CCDS early because, nowadays, a clinical antemortem diagnosis still does not exist. There are many reasons. First, not all veterinarians are informed about the existence of CCDS, and/or during physical and clinical evaluations, they may think that some clinical signs refer to one of the most common diseases affecting elderly patients and, second, owners’ economic possibilities often do not allow veterinarians to investigate the condition in depth.

Owners’ attention towards their pets plays another important role; unfortunately, behavioral changes are very often considered normal consequences of the aging process; so, owners do not ask veterinarians to investigate the reason (aging or disease) behind these changes. Furthermore, the diagnostic process is complex. What is nowadays commonly accepted is the exclusion of other possible pathological conditions whose clinical signs are comparable to those of CCDS. To achieve this goal, veterinarians collect blood samples for complete blood count and serum biochemistry, but it is not clear which parameters must be detected, and which are the limits between physiological and pathological. Theoretically, the more parameters are evaluated, the more possibilities to rule out other pathological conditions are in place. This is made more difficult when we consider more advanced examinations, such as ultrasound evaluation, MRI and CSF samples. The first one is a less invasive technique that can help us in the exclusion of other conditions, but ultrasound is not determinant in CCDS diagnosis, while MRI may be decisive for the comprehension of anatomical alterations compatible with behavioral changes. However, anesthesia is required, and it is an expensive exam. In addition, veterinarians are required to have an appropriate knowledge for the interpretation of images and MR scans are not yet widely present. The same considerations may be applied to CSF samples, which always require anesthesia and veterinary experience. Additionally, considering what is present in the literature, the results obtained by different authors are often in contrast or they are not comparable. In fact, different protocols have been used, different numbers of animals have been enrolled, and, in many cases, the same tools or procedures have been modified or adapted to various necessities.

Regarding cognitive tests, the situation is similar: there are a lot of different tasks which can be used to evaluate cognitive skills and the sensory system; so, authors have used different types of tests and in various modalities and combinations. Major difficulties may be represented by a scenario in which tests that should guarantee the absence of external stimuli (noises, smells, objects, and people) are performed, the fact that animals may be inhibited and may not have a natural behavior because of fear, or the subjectivity in the interpretations of results. All these factors together represent how difficult deciding which are the most appropriate cognitive tests to choose for a possible CCDS diagnosis is.

Scientists agree that CCDS is the counterpart of Alzheimer’s diseaseA One Health approach should permit both human and veterinary medicine to take many steps forward in the achievement of these goals. Neurodegenerative pathologies are very hard conditions to face both for patients and for family members. CCDS deteriorates the relationship between dogs and owners. In most of the cases, owners ask for veterinary intervention when they feel too tired and frustrated because they cannot manage domestic life with their pets. Unfortunately, when clinical and behavioral signs become so severe, it is too late: severe clinical manifestations are strictly related to severe neurodegeneration, which is a progressive and irreversible condition. This kind of situation leads to owners’ irritability and frustration, all elements which contribute to aggravate the relationship with their dogs, without considering that all these things also affect the animals’ welfare.

In conclusion, there are a lot of interesting works that seem to offer very promising tools (e.g., questionnaires, EEG recordings, MRI parameters, cognition tests, etc.) for a presumptive CCDS diagnosis [4,14,30,35,45,46,47,48,49] but, in the majority of the cases, the techniques used are too expensive or too difficult to reproduce for those who are not experts. For the (near) future, among the most important objectives in the management of CCDS are a better understanding of the pathogenesis and the identification of early diagnostic tests that can allow for a targeted, patient-oriented therapeutic approach. Secondarily, but not as regards importance, a better comprehension of CCDS may have many positive effects on human medicine, on the welfare of patients affected by Alzheimer’s disease, and on owners’ welfare in the coexistence with their pets in the domestic space.

## Figures and Tables

**Table 1:** Summary of the main different techniques and diagnostic protocols, and related results that have been proposed in last decades for antemortem diagnosis of CCDS.

Diagnostic Tool	References	Findings
**Clinical and neurological examinations**	[38]	-Correlation between decrease in frontal lobe volume and hippocampal region with declines in cognitive tests
	[39]	-Higher level of proteins in lightly and severely cognition-impaired dogs -High levels of glucose, pyruvate, potassium and lactate in severely cognition-impaired dogs (alteration in cerebral glucose metabolism)
	[26]	-Significant high levels of Aβ-42 in dogs suffering from cognitive impairment
	[40]	-No significant differences in vitamin E levels between non-impaired dogs and impaired ones
	[8]	-Significant increase in Aβ-42 levels in dogs with CCDS -No significant results concerning cytokines, fibrinogen and C-reactive protein levels -Olfaction decline as a possible indicator of CCDS
	[23]	-Lower blood concentrations of Aβ oligomers
	[41]	-Plasma NFL levels in dogs affected by CCDS were higher than those in healthy dogs
	[4]	-Noteworthy increase in alanine aminotransferase (ALT), gamma glutamyl transferase (GGT), total protein (TP) and decreased values of chloride and sodium in mildly cognition-impaired dogs -Remarkable increased level of NFL in mildly cognition-impaired dogs -No significant differences for tau proteins or for Aβ-42
	[31]	-Positive correlation between worse periodontal diseases and cognitive impairment
	[35]	-High levels of NFL were positively correlated with bad performances in working memory tasks and high CADES scores
	[30]	-Dogs with CCDS showed loss of gamma coherence, compatible with decreasing functional connection between hemispheres -Dogs at risk of developing CCDS showed higher alpha P3-P4 coherence than dogs affected by CCDS -Alpha interhemispheric coherence may be an early indicator of CCDS -Dogs with behavioral changes also showed alterations in their cerebral electrical activity
	[42]	-NFL plasma levels of CCD dogs were significantly higher than in healthy dogs -Liver and renal dysfunction biomarkers (AST, ALT, GGT, urea nitrogen, total protein, albumin, SDMA and creatinine) were increased in CCD dogs -Higher levels of C-reactive protein and low levels of sodium and chloride were found in CCD dogs -NFL blood concentrations increase during aging in dogs
	[34]	-Investigation of RBP4, CXCL10 and NOX4 in blood samples with ELISA kits -Decreased levels of RBP4, CXCL10 and NOX4 in dogs affected by CCDS compared to normal dogs
	[43]	-No significant results for hematic concentration of Aβ40, Aβ42 and GFAP, and Aβ42/Aβ40 ratio in dogs with presumptive CCDS -Strong and significant correlation with CCDS scores (investigated with three validated questionnaires: CADES, CCDR and CCAS) and increased levels of serum NfL
**Diagnostic Tool**	**References**	**Findings**
**Magnetic Resonance Imaging (MRI)**	[38]	-Total brain volume decreased with age -Remarkable increase in the anterior portion of lateral ventricle in senior dogs -Decreased frontal lobe volume was observed in dogs of more than 8 years of age -Decreased volume of different brain areas negatively influenced the dogs’ ability to perform size discrimination and reversal, spatial list learning, size-learning set discrimination, and concept-learning tests
	[44]	-Significant negative correlation between interthalamic adhesion thickness and age -Significant positive correlation with body weight -Interthalamic adhesion thickness as a possible good indicator of brain atrophy
	[45]	-The interthalamic adhesion thickness, the interthalamic adhesion thickness/brain height ratio and interthalamic adhesion thickness/brain height to lateral ventricle/brain height were significantly lower in cognition-impaired dogs
	[23]	-MRI showed diffuse cortical atrophy and white matter’s hyperintensity
	[5]	-Dogs affected by CCDS had smaller total hippocampal volumes compared to aged dogs without CCDS
	[16]	-Evaluation of BBB permeability using SEA technique on MRI -No significant differences in BBB permeability comparing healthy dogs with dogs affected by CCDS -Interthalamic adhesion diameters were smaller in dogs affected by CCDS
**Diagnostic Tool**	**References**	**Findings**
**Cognitive Tests/questionnaires**	[46]	-Mild visuospatial function decline was detected in dogs of 6 years of age: a possible sensitive sign of cognitive impairment prior to the onset of clinical signs
	[47]	-Problem-solving task was age-sensitive, with young dogs performing better -The food-searching task was sensitive to age and to CDS severity
	[8]	-Increased levels of Aβ-peptides were positively correlated with CCDR score -“Aimless wandering”, “staring blankly into space”, “avoiding being patted” and “difficulty with finding dropped food” were the most observed signs in dogs affected by CCDS
	[25]	-Dogs with high levels of Aβ-42 in the brain showed learning impairments in discrimination learning, reversal learning and Delayed Non-Match Position (in particular, the DNMP skill is affected early during aging)
	[48]	-Correlation between CCD and vision impairment, smell disturbances, tremor, swaying or falling, and head ptosis
	[18]	-No correlation between cognitive performances [Modified Vienna Canine Cognitive Battery (MVCCB)] and one year of diet rich in antioxidants -Problem solving is the factor most influenced by cognitive ability -Decline in sociability, playfulness, dependency and boldness paralleling with increasing age
	[41]	-Positive correlation between NFL increases and CADES scores
	[49]	-Impaired dogs showed worse performances in the food-searching task -Difficulty in finding dropped food is a commonly reported feature in dogs with moderate CCDS
	[35]	-CADES scores and CCDR scores showed different results, often discordant -Different results in the control task between healthy and impaired dogs -Strong relationship between sustained attention and CADES category -Performances in the inhibitory control task were highly associated with CADES category, CCDR category and NFL levels -Executive control (inhibitory control and detour) and sustained gaze test were the most significant fields affected by the aging process
	[30]	-Rofina score was correlated with alpha Fp1 power
	[14]	-CCAS was found to be a practical tool to assess cognitive impairment in domestic dogs -PCT performances were shown to be related to the aging process
	[34]	-Classification of dogs’ cognitive impairment through CCDR questionnaire
	[37]	-CADES and CCAS showed strong similarities and efficacy in the prediction of CCD from a mild to a more severe state of CCD -CCDR is more useful in dogs showing severe signs of CCD -C-BARQ can be a support in the investigation of mild cases of CCD, especially for the domains assessing fear
	[32]	-Evaluation of the correlation between body mass and cognitive impairment (finding no significant correlation) -All the tasks [five spontaneous problem-solving tests: two memory tasks (two-location task, delayed search task), a sensory screen, two measures of executive function (cylinder task, spatial reversal task) and a measure of human social interaction], except for social interaction, showed worse performances with increasing aging, especially the delayed search task and the spatial reversal task
**Diagnostic Tool**	**References**	**Findings**
	[23]	-High levels of Aβ oligomers in CSF, with a prevalence of the Aβ-42 type

**Table 2:** Most frequently used questionnaires and characteristics features.

Questionnaire	Most Relevant Features
CAnine DEmentia Scale (CADES)	It seems to give a better idea of the different stages of CCDS, to identify cognitive impairment earlier, and to monitor the progression of this disease over time if compared with CCDR. The combination of the CADES questionnaire and the evaluation of plasma biomarkers may be better at predicting CCDS in its early stages.
Canine Cognitive Dysfunction Rating scale (CCDR)	CCDR was designed to find a clinically and ethologically valuable screening tool for CCDS diagnosis. Even if the diagnostic accuracy of CCDR scale is high (~98.9%), it seems to be more reliable when behavioral alterations are severe. It is more focused on the frequency of the behavioral alterations.
By Kiatipattanasakul et al. [52]	It includes items about posture alterations.
By Ozawa et al. [48]	It is divided into three sections about general information, physical disturbances and the CCDR. The results showed a correlation between physical disturbances and CCDR, especially for vision impairment (present in more than 90% of CCDS dogs), according to human medicine, where ocular tests could be used as potential biomarkers to early diagnosis of AD.
Canine Cognitive Assessment Scale (CCAS)	It is a questionnaire that has been modified from others already known in the literature; it is divided into 6 groups (Disorientation, Sleep–wake cycle, Anxiety, Activity level, Learning and memory, and Social interactions), which include 17 items to be addressed by owners on a 0 (never) to 3 (almost every day) scoring system.
Practical Cognitive Test (PCT)	It can be used with dogs not living in a home environment. It consists of two tasks: discrimination learning and reversal learning.

## Data Availability

No new data were created or analyzed in this study. Data sharing is not applicable to this article.

## Abbreviations

The following abbreviations are used in this manuscript:

AD	Alzheimer’s Disease
ALT	Alanine aminotransferase
AST	Aspartate aminotransferase
Aβ	Amyloid βeta
BBB	Blood–Brain Barrier
C-BARQ	Canine Behavioral Assessment and Research Questionnaire
CADES	Canine Dementia Scale
CCAS	Canine Cognitive Assessment Scale
CCDR	Canine Cognitive Dysfunction Rating scale
CCDS	Canine Cognitive Dysfunction Syndrome
CSF	Cerebrospinal fluid
CT	Computed Tomography
CXCL10	C-X-C-motif chemokine ligand 10
DISHA(A)	Disorientation, social Interactions, Sleep-wake cycle, House-soiling, Activity level (Anxiety/Aggressivity)
DNMP	Delayed Non-Match Position
EEG	Electroencephalogram
GABA	Gamma-aminobutyric acid
GFAP	Glial Fibrillary Acidic Protein
GGT	Gamma glutamyl transferase
HPLC	High-Performance Liquid Chromatography
IA	Interthalamic Adhesion
JLZC	Joint Lempel–Ziv Complexity
MRI	Magnetic Resonance Imaging
MVCCB	Modified version of Vienna Canine Cognitive Battery
NfL/NFLs	Neurofilament light chain/s
NFTs	Neurofibrillary Tangles
NOX 4	NADPH oxidase 4
PCT	Practical Cognitive Test
PT	Total Protein
RBP4	Retinol-binding protein 4
RDS	Rofina Dysfunction Score
SDM	Symmetrical Dimethylarginine
SEA	Subtraction Enhancement Analysis
UV	Ultraviolet
